# Isolation of peste des petits ruminants virus using primary goat kidney cell culture from kidneys obtained at slaughter

**DOI:** 10.1002/vms3.413

**Published:** 2020-12-16

**Authors:** Shahana Begum, Mohammed Nooruzzaman, Azmary Hasnat, Mst. Murshida Parvin, Rokshana Parvin, Mohammad Rafiqul Islam, Emdadul Haque Chowdhury

**Affiliations:** ^1^ Department of Pathology Faculty of Veterinary Science Bangladesh Agricultural University Mymensingh Bangladesh

**Keywords:** Black Bengal goats, goat kidney cell culture, *peste des petits ruminants*, virus isolation

## Abstract

**Background:**

Traditionally isolation of *peste des petits ruminant* virus (PPRV) is performed in Vero cells that takes several blind passages before observing typical cytopathic effects (CPEs). As an alternate, researchers have been using lamb kidney (LK) cells but day‐old lambs are difficult to obtain and requires animal sacrifice.

**Objective:**

We established a primary goat kidney (GK) cell culture from the kidneys obtained at slaughter.

**Methods:**

The kidney of Black Bengal goats were collected from slaughter house and processed to make single cell suspension. The cells were resuspended in appropriate culture medium and maintained under optimum culture condition.

**Results:**

The 80% confluent monolayer of GK cells was obtained after 15–20 days post seeding. Upon infection with a field isolate of PPRV, the well‐developed CPEs characterized by cell rounding, vacuolation in the cytoplasm and fusion of cells were observed after 48 hr post infection. Virus quantification in the culture supernatant revealed more viral RNA in GK cells than LK cells. The multicycle growth analysis of PPRV showed a steady increase in the virus loads in the culture supernatant of infected GK cells, suggesting an adaptation of the PPRV in GK cells.

**Conclusions:**

The findings suggest that primary GK cells can be successfully prepared from the mature kidney cortical tissues and can be used for the isolation of PPRV. This system could reduce the unnecessary sacrifice of lambs or kids. Since kidneys of slaughtered goats are available throughout the year, using this protocol primary cell culture from mature goat kidney can provide primary cells to the laboratory throughout the year.

## Graphical abstract

We established a primary goat kidney cell culture system using kidneys of adult goats obtained at slaughter. The developed cell culture system allows efficient replication of field strain of peste des petits ruminants virus (PPRV). Therefore, primary goat kidney cells can be successfully prepared from the mature kidney cortical tissues and can be used for the isolation of PPRV.

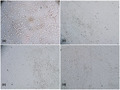

## 1 INTRODUCTION

*Peste des petitis ruminants* (PPR) is one of the most contagious and fatal disease of small ruminants primarily of sheep and goats and is currently endemic in most parts of Asia, Africa and the Middle East. The causative agent PPR virus (PPRV) is a member of the genus *Morbillivirus* under the subfamily *Paramyxovirinae*, the family *Paramyxoviridae* and the order *Mononegavirales* (Gibbs et al., 1979). There are seven known and antigenically closely related members of the genus *Morbillivirus*: measles virus (MV), rinderpest virus (RPV), PPRV, canine distemper virus (CDV), cetacean morbillivirus (CeMV), phocine distempervirus (PDV) and feline morbillivirus (FMV) (Kumar et al., 2014).

Several cell lines and primary cell cultures have been used for the isolation and propagation of PPRV *in vitro*. Cell lines that are sensitive to PPRV include Madin Darby Bovine Kidney (MDBK), Baby Hamster kidney clone 21 (BHK‐21), normal African green monkey kidney fibroblast cells (CV1) and particularly the African green monkey kidney (Vero) cell line (Silva et al., 2008). The Vero cell line has been commonly used for the isolation and cultivation of morbilliviruses including PPRV and for vaccine production (Nanda et al., 1996). However, the isolation of PPRV using Vero cells usually requires several blind passages before observing the PPRV‐induced cytopathic effects (CPE), and the CPE is sometime difficult to detect (Kumar et al., 2016; Sreenivasa et al., 2006). Moreover passaging of PPRV in Vero cells leads to the attenuation of the virus in susceptible hosts upon experimental infection (Pope et al., 2013; Truong et al., 2014).

A remarkable development in cell culture system of PPRV happened with the discovery of two cell surface receptors of morbilliviruses. The signalling lymphocyte activation molecule (SLAM) also known as CD150, a protein receptor expressed primarily on the lymphoid cell surface, is used preferentially by wild‐type morbilliviruses to bind to the host (Tatsuo et al., 2001). Another receptor, the Nectin‐4 has also been showed as putative receptor of morbilliviruses in epithelial cell surface (Noyce et al., 2011). The overexpression of recombinant SLAM or Nectin‐4 in cell lines showed efficient replication of morbilliviruses in cell lines that showed limited replication previously. For example, the Vero/SLAM showed efficient replication of wild strains of CDV (Seki et al., 2003). Similarly, the monkey kidney cells CV1 overexpressing goat SLAM showed efficient replication of PPRV from pathological specimens (Adombi et al., 2011). The overexpression of Nectin‐4 also permitted efficient replication of PPRV in epithelial cells (Birch et al., 2013; Fakri et al., 2016). Beside kidney cells, lymphoid cell line (B95a) from marmoset, which carry the SLAM receptor on cell surface, were also shown to be very efficient in propagating PPRV (Sreenivasa et al., 2006). To our experiences, the limitation of using these recombinant cell lines in developing countries is transportation and maintenance of the cells using cool chain.

PPRV isolate losses its pathogenicity due to propagation in the unusual host cells (Adu et al., 1990). But pathogenesis study through experimental infection requires viruses with unaltered pathogenicity. Isolation of PPRV using primary lamb and goat kidney cell cultures could eliminate some of the limitations of cell line. The characteristics CPE in primary goat and lamb kidney cell cultures can be found during the one to two passages of virus infection and the pathogenicity of the propagated virus was found unaltered (Govindarajan et al., 2008; Taylor & Abegunde, 1979). However, kidneys of 1 to 2 weeks old lambs for the preparation of primary cell culture are often hard to obtain and require animal sacrifice. Therefore, we established primary goat kidney cell culture from kidneys of adult goats obtained at abattoir and successfully isolated PPRV using primary goat kidney cell culture.

## 2 MATERIALS AND METHODS

### 2.1 Preparation of primary goat kidney cell culture

Primary goat kidney cell culture was prepared using mature goat kidneys collected from slaughtered goats according to the procedure described by FAO (Rweyemamu, 1994) with minor modifications. Briefly, fresh kidneys from adult goats were collected at slaughter, transferred into a sterile glass jar containing ice and transported immediately to the laboratory. The kidney was washed thrice with phosphate buffered saline (PBS). Then the fascia and capsule were removed and the kidney was washed thrice again in PBS. The cortical tissues were collected in a glass petri dish and cut into small pieces using sterile scissors and washed several times in PBS. The small pieces of cortical tissues were further minced into small pieces and washed again. The minced kidney tissues were trypsinized for 20 min at room temperature using 0.25% trypsin (Gibco, Cat: 15090046) under constant stirring. The supernatant of first trypsinization was discarded. Subsequent trypsinization was performed for 30 min at room temperature. The supernatant of the cell suspension was harvested in 10 ml of foetal calf serum (FCS) and stored at 4°C. The supernatants of such two or three preparations were pooled and centrifuged at 800 rpm for 10 min at 4°C. The cell pellet was resuspended in growth medium (M‐199 medium containing 15% foetal calf serum (FCS), 0.1 mg/ml Gentamicin and 250 µg/ml Fungizone) and the final volume was adjusted to obtain 2 million cells per ml of the culture medium. Finally, 5 ml of diluted cell suspension was dispensed in 25 cm^2^ culture flask and incubated at 37°C with 5% CO_2_. The cells were monitored daily under inverted microscope. After 7 days, the growth medium was changed with similar growth medium with required additives. Simultaneously, the lamb kidney cells were also prepared following the above mentioned protocol.

### 2.2 Subculture of primary kidney cells and infection with local a PPRV isolate

The monolayer of first passage of primary goat kidney cell culture at about 80% confluency was trypsinized using 0.25% Trypsin (Gibco, Cat: 15090046)‐Ethylenediaminetetraacetic acid (EDTA) solution. Cells were suspended at 1:3 ratios with the growth medium containing 12% FCS and other additives and dispersed into new culture flasks. At 80% confluency, cells were trypsinized as above, resuspended and used for next passages. Five of such subcultures were performed consecutively. For the isolation of PPRV, the 80% confluent monolayer of goat kidney cells was used. Initially, a 20% lymph node homogenate (RT‐PCR confirmed for PPRV) obtained from natural PPR infected goats was used as inoculum. The culture supernatant was removed and the cells were washed with warmed PBS twice. Then 100 µl inoculum was added to the culture flask and incubated for 1 hr at 37°C. PBS was used as mock infection in the control flask. Then culture medium containing 5% FCS was added to the flask and incubated again. The cells were monitored twice daily under microscope for the development of CPE. The effects of carryover toxins, if any, present in inoculum was monitored up to 48 hr by observing cell morphology. The infected cells were frozen and thawed 5 times when maximum CPE were observed at 96 hpi. Then the culture fluid was centrifuged at 1,200 *g* for 15 min. The supernatant was collected, divided into small aliquots and stored frozen (−80°C) until used. For consecutive passages, the cell culture monolayers was prepared and inoculated with the virus suspension using above mentioned frozen inoculum. When maximum CPE manifested, the virus suspension was harvested and stored as above. The serial passaging of the virus in primary goat kidney cell culture was continued until the 3rd passage and the quantification of virus titre in the culture supernatant was performed using 96‐well flat bottom tissue culture plates as per description of Reed and Muench (Reed & Muench, 1938). Similar infection protocol was also performed for primary lamb kidney cell culture. For multicycle growth analysis, confluent monolayers of primary goat kidney cells were infected with PPRV as above and the culture supernatants were harvested at 24, 36, 48, 60, 72 and 96 hpi and the amount of PPRV RNA in the culture supernatants was quantified by real time quantitative RT‐PCR (RT‐qPCR).

### 2.3 Culture of goat kidney cells on coverslip

A sterile plastic petri dish containing glass coverslip was used to propagate the primary goat kidney cells. The confluent monolayer of goat kidney cells was harvested using 0.25% trypsin and resuspended in M‐199 medium containing 12% FCS. About 4 ml (one million cells/ml) of cell suspension was dispensed into petri dishes and incubated at 37°C in a CO_2_ incubator. Upon confluency, 100 µl of the PPRV was inoculated into the goat kidney cells and observed for the development of the PPRV‐induced CPE. When approximately 80% of the cells developed CPE, the cells on coverslip were fixed with ice‐cold methanol and stained with haematoxylin and eosin (H&E) stain as per standard method.

### 2.4 Detection and quantification of PPRV

Viral RNA was extracted from both cell lysates and culture supernatants using PureLink™ Viral RNA/DNA Mini Kit (Invitrogen). The presence of PPRV RNA in the cell lysates was detected by an established conventional RT‐PCR method (Forsyth & Barrett, 1995). A 448 bp fragment of F gene of PPRV was amplified using Superscript III OneStep RT‐PCR kit (Invitrogen) as per the manufacturer's instructions. The quantity of the PPRV RNA in the cell lysates and supernatants was determined by RT‐qPCR method using Rev Trans QPCR One‐Step EvaGreen® (ROX) (Bio&SELL, Germany). For RT‐qPCR, the forward primer NP3 (5ʹ‐TCTCGGAAATCGCCTCACAGACTG‐3ʹ) and reverse primer NP4 (5ʹ‐CCTCCTCCTGGTCCTCCAGAATCT‐3ʹ) was used as described by Couacy‐Hymann and colleagues (Couacy‐Hymann et al., 2002). For internal control of RNA input, equal amount (20 ng/reaction) of RNA was used for all samples. The result was analysed by ΔCt (delta cycle thresold) method using the following formula: ΔCt = (*Ct* target‐*Ct* uninfected control). The change in the viral gene expression was calculated as 2^‐ΔCt^ and the value indicated in an n‐fold difference relative to the uninfected control.

## 3 RESULTS

### 3.1 Establishment of primary goat kidney cell culture

**FIGURE 1:**
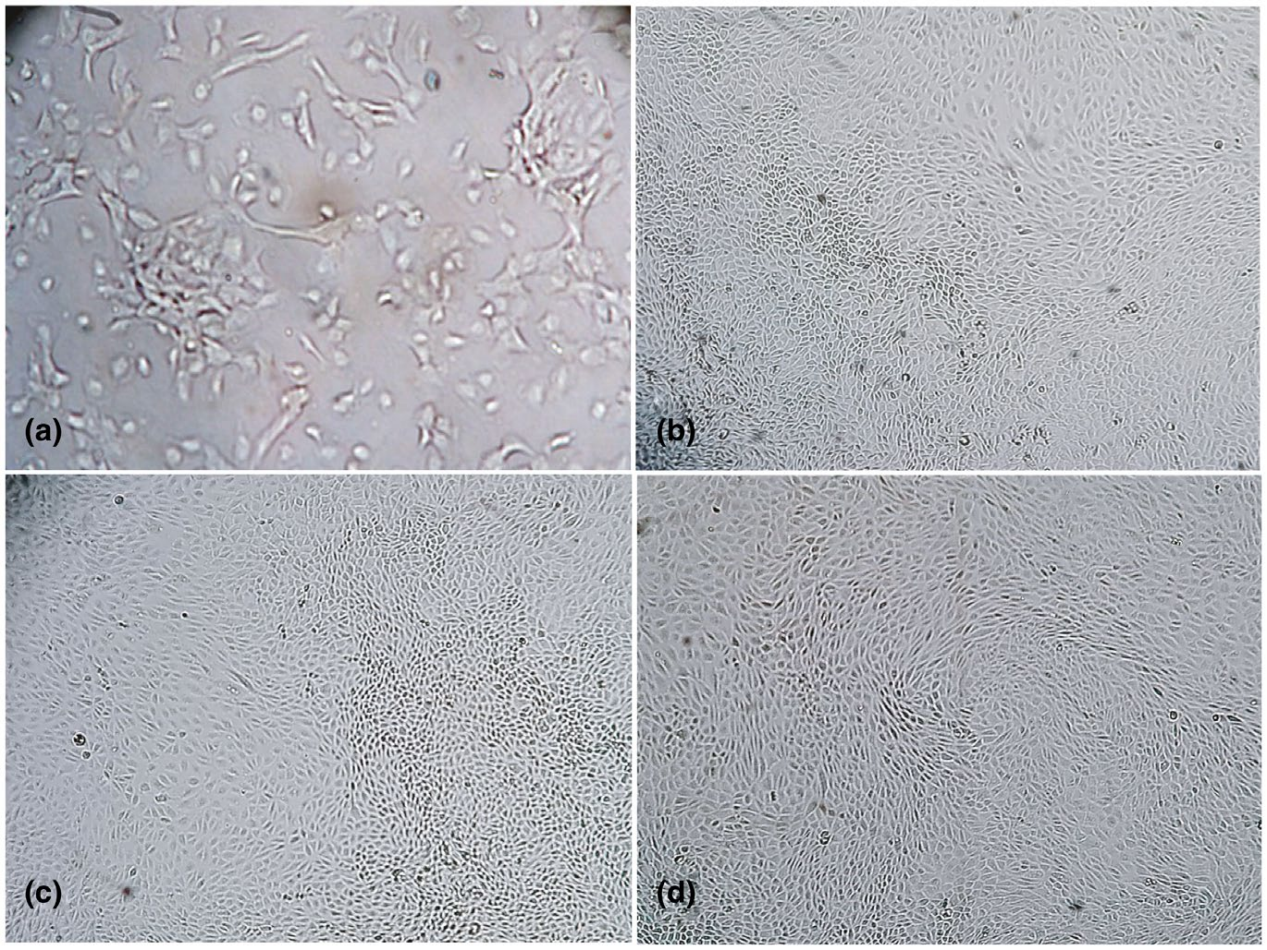
Primary goat kidney cell culture from kidneys obtained at slaughter. Growing goat kidney cells in culture at day 7 (a), 80% confluent monolayer at day 15 (b), confluent monolayer at day 20 (c) and over grown cells at day 30 (d). Objectives: 20× (a), 10× (b–d)

The goat kidney cells settled down and started to grow after 4–5 days of seeding and became morphologically distinct after 7 days (Figure 1a). The 80% confluent monolayer of primary goat kidney cells was obtained after 15 days post seeding (Figure 1b) and full confluency was found at day 20 post seeding (Figure 1c). The primary goat kidney cells were maintained up to 30 days post seeding although the cells were overgrown and appeared to die (Figure 1d). However, the lamb kidney cells took about 15 days for full confluency (data not shown). During the 2nd and 3rd subculture, goat kidney cells grew relatively faster than the 1st culture and became confluent by day 10 post seeding. However, the growth rate of primary goat kidney cells decreased from the 4th subculture, although the cell morphology remained unchanged.

### 3.2 Infection morphology of PPRV in primary goat kidney cells

**FIGURE 2:**
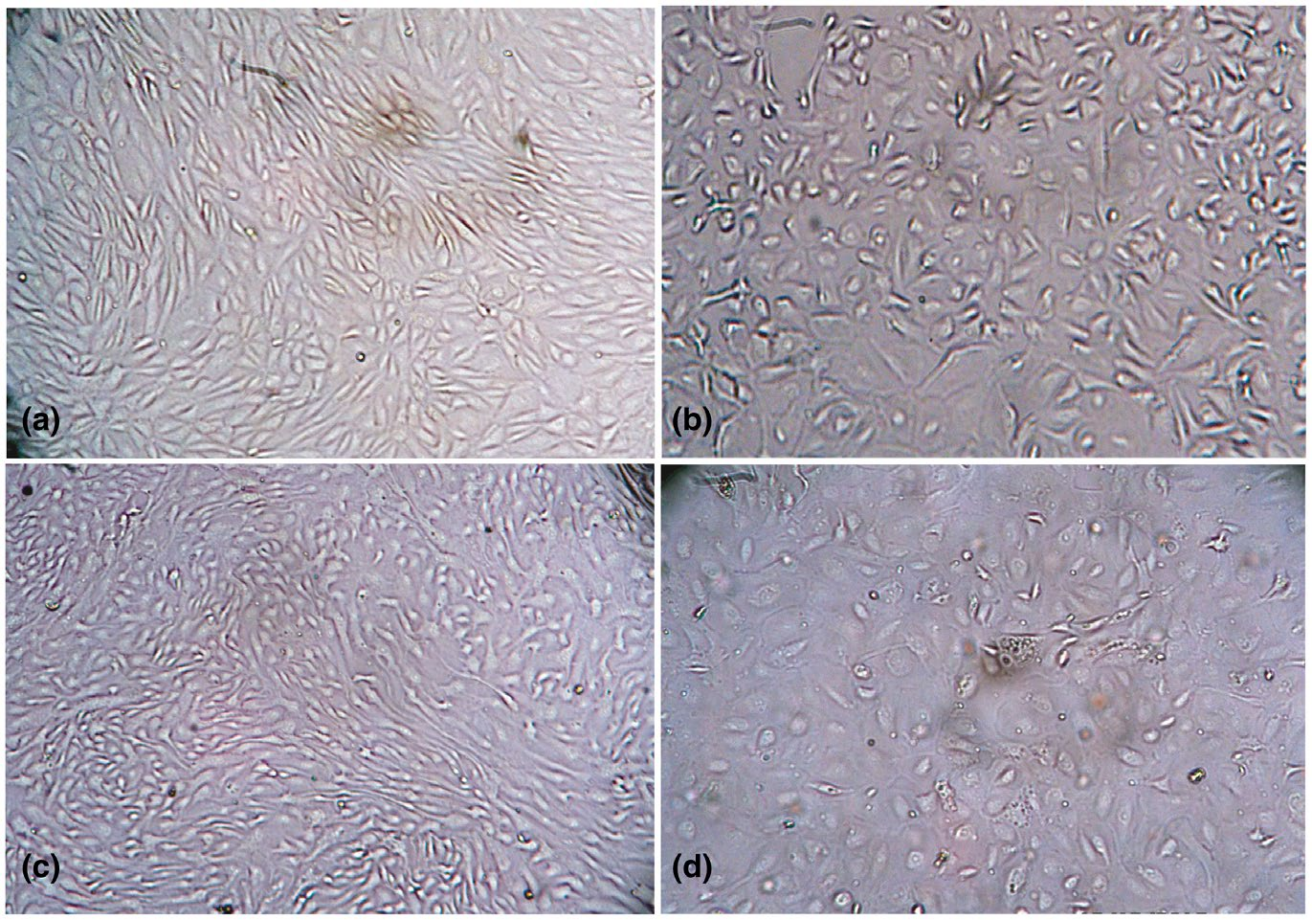
Morphology of PPRV infected primary goat kidney cells at 36 and 48 hr post infection. Uninfected normal (a) and infected goat kidney cells at 36 hr post infection showing rounding of cells (b), uninfected normal (c) and infected goat kidney cells at 48 hr post infection showing vacuolation and syncytia formation (d). Objectives: 20× (a–d)

During the first passage, the initial CPE characterized by cell rounding started to appear at 48 hr post infection (hpi). During 2nd and 3rd passages, initial cell rounding of limited extent was observed a bit earlier at 36 hpi (Figure 2b). However, the well‐developed CPE characterized by cell rounding, vacuolation in the cytoplasm and fusion of cells were observed at 48 hpi (Figure 2d). The mock infected cells during all time points remained normal (Figure 2a,c).

**FIGURE 3:**
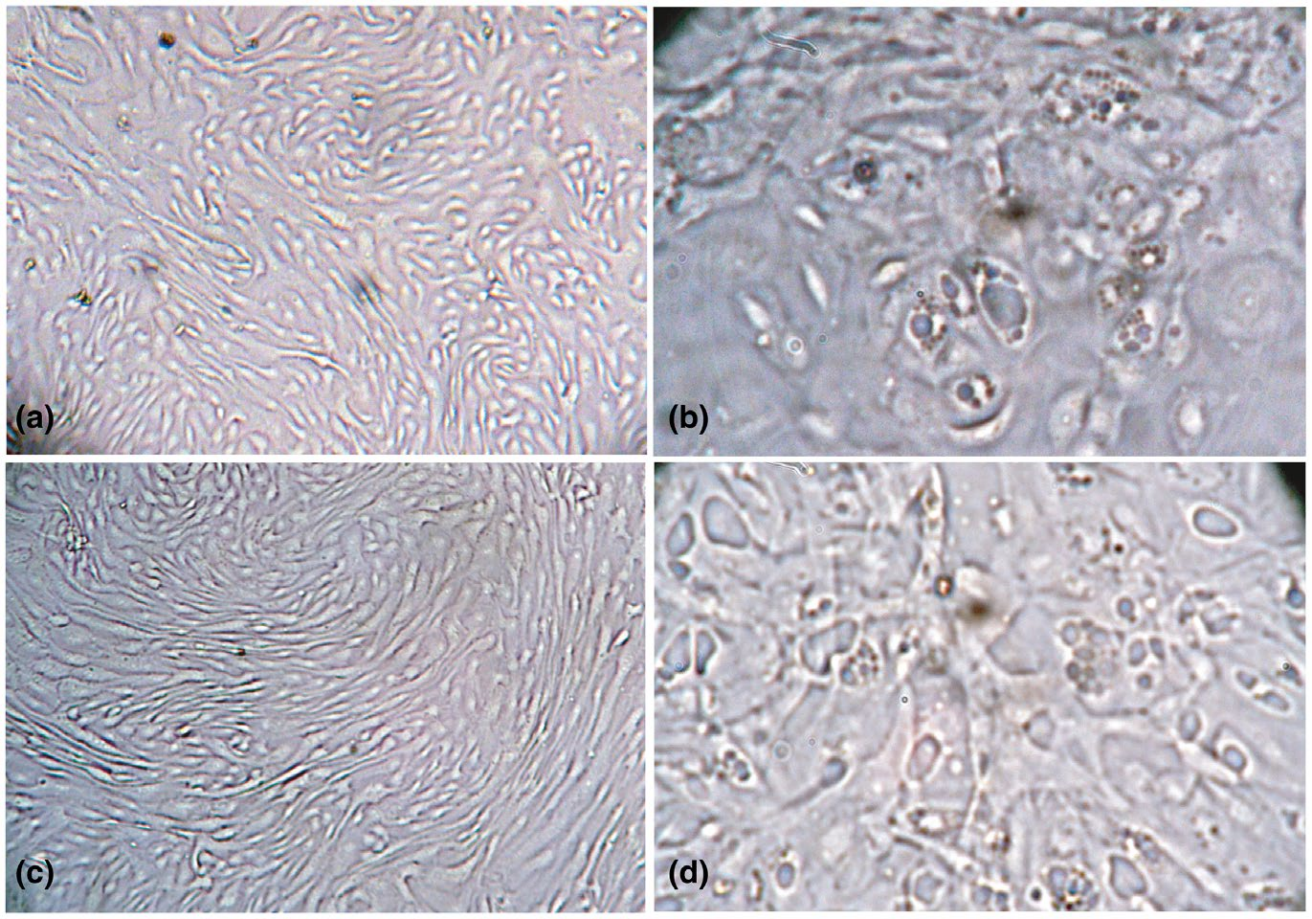
Morphology of PPRV infected primary goat kidney cells at 72 and 96 hr post infection. Uninfected goat kidney cells at 72 (a) and 96 (c) hours post infection showing normal morphology. PPRV infected goat kidney cells showing vacuolation, syncytia formation and nuclear budding at 72 (b) and 96 (d) hours post infection. Objectives: 20× (a, c), 40× (b, d)

With times, the infected cell retracted and syncytia were formed at 72 hpi (Figure 3b). The vacuolation and syncytia formation became distinct at 96 hpi during which more than 80% of the goat kidney cells were infected (Figure 3d).

**FIGURE 4:**
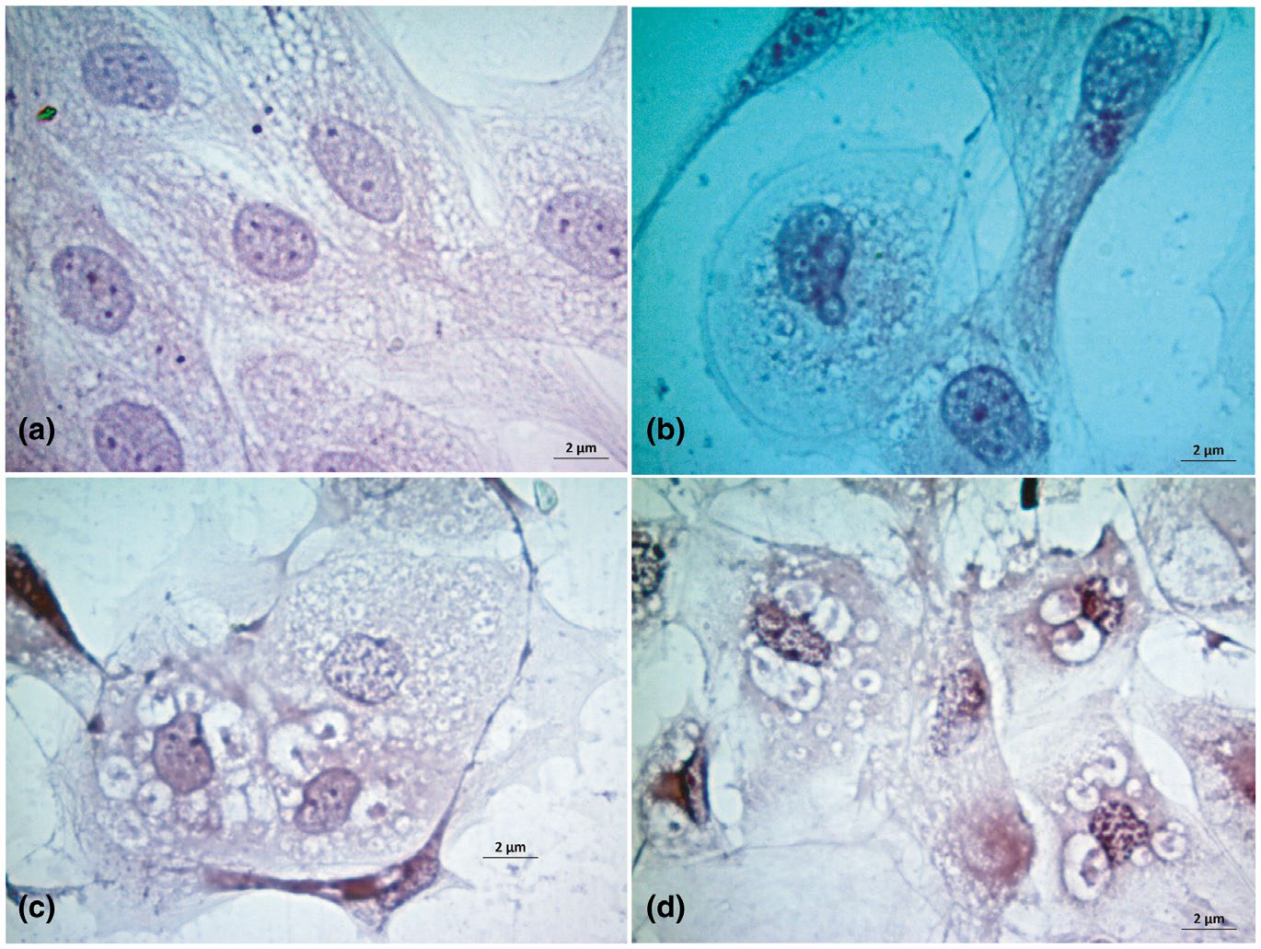
Study of cytopathic effects in goat kidney cells upon H & E stain. Uninfected control goat kidney cells (a), nuclear budding with syncytia formation at 48 hr post infection (b), vacuolation, nuclear budding and syncytia formation at 72 hr post infection (c) and vacuole, syncytia and nuclear budding at 96 hr post infection in goat kidney cells (d) are shown. Bar (=2 µm) indicates the magnification

We also examined the morphology of the PPRV‐induced CPE in goat kidney cells using H&E stain. The mock infected goat kidney cells were found morphologically homogenous with almost spherical nuclei (Figure 4a). After infection of PPRV, nuclear rounding with budding and small vacuolation started in the cytoplasm at 48 hpi (Figure 4b). With times, cellular fusion with increase number and sizes of the cytoplasmic vacuoles at 72 hpi (Figure 4c), syncytia formation and lysis of cells at 96 hpi were observed (Figure 4d–f).

### 3.3 Quantification of PPRV in the primary goat kidney cell culture

**FIGURE 5:**
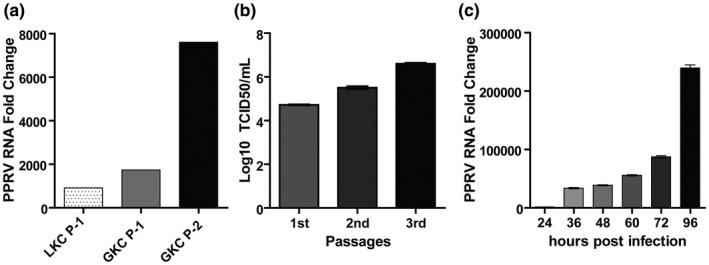
Quantification of PPRV in infected primary goat and lamb kidney cells. (a) PPRV loads expressed as fold change (2^−ΔCt^) over infected control in the supernatant of primary goat and lamb kidney cell cultures. Here, LKC‐P1: Lamb kidney cells passage 1, GKC‐P1 and P2: Goat kidney cells passage 1 and 2. (b) Titration of PPRV in the supernatant of infected primary goat kidney cells during three consecutive passages by TCID_50_ method. (c) PPRV RNA expressed as fold change (2^−ΔCt^) over uninfected control in the supernatant of primary goat kidney cells infected with PPRV at different time post infection. Data indicate mean ± *SEM* (b–c)

Replication of PPRV in primary goat kidney cells was confirmed by RT‐PCR. The supernatant of PPRV‐infected primary goat and lamb kidney cell culture fluids were collected at 96 hpi following repeated freeze‐thawing cycle and centrifugation and was tested for the presence of PPRV RNA by RT‐PCR. All culture supernatants from infected goat and lamb kidney cells showed positive amplification during RT‐PCR. When we compared the virus replication in lamb kidney cells and goat kidney cells, we found more viral RNA in goat kidney cells than the lamb kidney cells as shown by higher fold change in goat kidney cells than lamb kidney cells (Figure 5a). Next, we performed three consecutive passages of PPRV in primary goat kidney cells and the amount of PPRV in the culture supernatant from all three passages was quantified by TCID_50_ method. The primary goat kidney cells showed optimum growth of PPRV as indicated by a steady increase in the titre of virus in the culture supernatants from 4.71805 log 10 TCID_50_/ml in 1st passage to 5.5035 log 10 TCID_50_/ml and 6.60505 log 10 TCID_50_/ml in 2nd and 3rd passages, respectively (Figure 5b). Finally we performed the multicycle growth analysis of PPRV in primary goat kidney cells. To this end, confluent monolayer of primary goat kidney cells were infected with PPRV inoculum as described earlier. The culture supernatant were harvested at 24, 36, 48, 60, 72 and 96 hpi and the amount of PPRV RNA in the culture supernatants was quantified by RT‐qPCR. We observed a steady increase in the viral RNA fold change in the culture supernatant over the time period and the highest amount of viral RNA was detected at 96 hpi (Figure 5c).

## 4 DISCUSSION

Primary goat kidney cell culture using mature goat kidneys obtained at slaughter was established successfully and the cells were passaged up to four subcultures. Goat kidney cells were readily allowed to infect with field isolates of PPRV. The characteristic CPEs such as cell rounding, vacuole formation in the cytoplasm and syncytia formation was obtained within 96 hpi.

In this study, the primary goat kidney cells produce about 80% confluent monolayer at 15 days post seeding and the full confluency was observed at 20 days post seeding. Lamb kidney cells took relatively shorter duration (15 days) for full confluency. Such differences could be due to the species and age differences as the kidney cells from lamb were collected from kid at sacrifice (Govindarajan et al., 2008). However, the subsequent passages of goat kidney cells took shorter duration (10 days) to become confluent which could be explained by the juvenile nature of the cells once they divide. The early cell rounding in goat kidney cells was visible after 36 hpi whereas it took about 48 hpi for lamb kidney cells (data now shown). The kinetics of PPRV‐induced CPEs depend on multiple factors such as host species, cells types, genetic differences of virus strains, etc. Several studies reported increase susceptibility of goats to PPRV infection than sheep (Fakri et al., 2017; Wernike et al., 2014). PPRV shows CPE as early as 2 days post infection in marmoset B lymphoblastoid cells (Sreenivasa et al., 2006). The influence of virus strains on cell culture permissiveness was also shown in another study where a locally circulating PPRV‐AR/87 strain showed very rapid onset of CPEs (18 hpi) as compared to the vaccine strain PPRV‐Sungri/96 (72 hpi) (Singh et al., 2010). The early CPEs found in PPRV‐infected primary goat kidney cells in this study could be explained by the origin of cells and characteristics of virus strain used. Natural PPR affected Black Bengal goats showed severe pathological changes in kidneys and elevated serum creatine kinase (Begum et al., 2018), and large amount of PPRV was detected in kidneys of Black Bengal goats experimentally infected with a local PPRV strain (Begum et al., 2020), suggesting an increase permissiveness of goat kidney cells to PPRV infection. Moreover the PPRV strain used in this study was collected from a local field outbreak in Black Bengal goat with severe lesions in kidneys. It suggests a high adoption of PPRV in kidney cells of Black Bengal goats. In line with this, we also found a relatively higher quantity of viral RNA in the supernatant of goat kidney cells. The multicycle growth analysis of PPRV in the primary goat kidney cells also showed a steady increase in the PPRV in the culture supernatant with the increase of time and passages. The characteristics CPEs induced by PPRV found in this study have also been described elsewhere (Adu et al., 1990; Saeed et al., 2004). The developed primary kidney cells are being routinely used for the isolation of field strains of PPRV in our laboratory and the pathogenicity of the propagated virus remained unaltered during subsequent experimental infection (Begum et al., 2020).

For the isolation of PPRV, kidneys cell lines such as MDBK, BHK‐21, Vero and CV1 have been used with some limitations. However, the cell lines overexpressing the SLAM or Nectin‐4 have many advantages over conventional cell lines and greatly enhanced the replication of PPRV in cell culture (Adombi et al., 2011; Birch et al., 2013; Fakri et al., 2016; Seki et al., 2003). The primary goat kidney cells developed in this study also showed efficient replication of field strains of PPRV. Therefore, the primary goat kidney cells can be readily used where the SLAM or Nectin‐4 expressing cell lines are not available.

## 5 CONCLUSIONS

In conclusion, we established a primary goat kidney cell culture using mature goat kidney cells which showed acceptable replication efficiency for PPRV. Such method can be applied for the isolation of PPRV from field outbreaks and fit well for the preparation of PPRV inoculum for experimental infection study. Furthermore, since kidneys of slaughtered goats are available throughout the year, it can be suggested that using this protocol primary cell culture from mature goat kidney can provide primary cells to the laboratory throughout the year. This could reduce the unnecessary sacrifice of lambs or kids.

## 6 ETHICS STATEMENT

All applicable national and institutional guidelines for the care and use of animals were followed. The study was carried out in accordance with the recommendation of the “Ethical Standard of Research Committee” of Bangladesh Agricultural University, Mymensingh. The protocol and procedures employed were reviewed and approved by the “Ethical Standard of Research Committee” (Ref. No. BAURES/ESRC/699/2020; Dated: 28.06.2020). The authors also confirm that the ethical policies of the journal, as noted on the journal's author guidelines page, have been adhered to and the appropriate ethical review committee approval has been received. The US National Research Council's guidelines for the Care and Use of Laboratory Animals were followed.

## CONFLICT OF INTERESTS

None declared.

## AUTHOR CONTRIBUTIONS

Shahana Begum and Mohammed Nooruzzaman contributed to methodology, software, validation, formal analysis, investigation, data curation, visualization, writing – original draft preparation, writing‐review and editing. Azmary Hasnat and Mst. Murshida Parvin contributed to methodology, validation and investigation. Rokshana Parvin contributed to methodology, investigation writing‐review and editing. Mohammad Rafiqul Islam contributed to investigation, formal analysis, writing – Review and Editing. Emdadul Haque Chowdhury contributed to conceptualization, formal analysis, investigation, data curation, writing – original draft preparation, writing – review and editing, supervision, project administration, funding acquisition. All authors have read and agreed to the published version of the manuscript.

## AUTHOR CONTRIBUTION

Shahana Begum: Data curation; Formal analysis; Investigation; Methodology; Software; Validation; Visualization; Writing‐original draft; Writing‐review & editing. Mohammed Nooruzzaman: Data curation; Formal analysis; Investigation; Methodology; Software; Validation; Visualization; Writing‐original draft; Writing‐review & editing. Azmary Hasnat: Investigation; Methodology; Validation. Mst. Mushida Parvin: Investigation; Methodology; Validation. Rokshana Parvin: Investigation; Methodology; Writing‐review & editing. Mohammad Rafiqul Islam: Formal analysis; Investigation; Writing‐review & editing. Emdadul Haque Chowdhury: Conceptualization; Data curation; Formal analysis; Funding acquisition; Investigation; Project administration; Resources; Supervision; Writing‐original draft; Writing‐review & editing.

### Peer Review

The peer review history for this article is available at https://publons.com/publon/10.1002/vms3.413.
